# A Biomimetic Four-Chamber Soft Actuator for Human-like Dexterous Manipulation with Spatial Bending and Twisting Capabilities

**DOI:** 10.3390/biomimetics11060360

**Published:** 2026-05-22

**Authors:** Yumeng Yin, Jiabin Yang, Fengyi Yuan, Gang Chen

**Affiliations:** School of Mechanical Engineering, Jiaxing University, Jiaxing 314000, China

**Keywords:** soft actuator, spatial bending, principle of virtual work, Multi-Chamber Structure, Human-like Finger, circular twisting

## Abstract

To address the challenge that existing soft grippers have difficulty achieving fine manipulation comparable to the human finger’s “circular twisting” motion, this paper proposes a four-chamber spatial bending soft actuator based on the principle of virtual work. The actuator incorporates an internal cross-shaped restricting layer that divides its cross-section into four independent pneumatic chambers. Through independent regulation of the pressure in each chamber, continuous and controllable bending in arbitrary spatial directions is achieved, replicating the bending and abduction/adduction degrees of freedom (DoFs) of a human finger and their composite motions on a single actuator. Based on the Yeoh hyperelastic constitutive model and the principle of virtual work, a static deformation model of the actuator is established. By introducing an engineering assumption of “deformation vector superposition” and correction coefficients fitted from experimental data, high-precision prediction from multi-chamber pressure input to spatial bending output is realized. Furthermore, a three-finger soft gripper is constructed based on this actuator, successfully demonstrating fingertip pinching and enveloping grasping. Through open-loop programmed control, the fine “circular twisting” manipulation is demonstrated (exemplified by light bulb installation). This study provides an effective structural design and modeling method for soft actuators to achieve decoupled multi-DoF motion control, showcasing their application potential in adaptability and dexterous manipulation.

## 1. Introduction

Soft robots demonstrate significant potential in fields such as precision grasping, medical rehabilitation, and exploration in confined spaces due to their inherent compliance, safe human–robot interaction capability, and excellent adaptability to irregular objects [1,2,3]. As a core actuating component of soft robots, pneumatic soft actuators have attracted considerable attention owing to their high power density, fast response, and design flexibility [4,5,6].

Early research, such as the pneumatic network theory proposed by Harvard University in 2011 and the development of a starfish-inspired soft gripper [7], laid the foundation for generating large bending deformation through chamber inflation. In 2013, Bobak et al. [8] addressed the issue of small deformation amplitude in silicone soft actuators by proposing that maintaining certain gaps between pneumatic network chambers could significantly increase deformation magnitude and rate. Since then, pneumatic network layouts combined with inter-chamber gaps have dominated soft robot design and fabrication [9,10,11]. With the advancement of 3D printing technology, mold manufacturing has become simpler and more convenient, greatly accelerating the design and fabrication speed of soft actuators using casting methods [12]. Furthermore, more numerous and complex internal chamber structures can be designed to achieve multiple deformation modes on a single actuator [13,14]. The research focus has shifted from achieving single motion patterns to endowing actuators with richer motion degrees of freedom. For instance, Zhu et al. [15] designed a multi-degree-of-freedom actuator capable of extension and bidirectional bending, integrating it into a crawling robot demonstrating mobility in constrained environments like pipelines. HUANG et al. [16] designed an Omnidirectional Bending Actuator capable of obstacle-climbing and inchworm-like crawling. Liu and Wang [17] designed a two-segment symmetric oblique-chamber actuator, achieving independent helical deformations in two segments through independent control of four air chambers, significantly enhancing the actuator’s adaptability to complex target shapes. These developments reflect the trend from single functionality to multi-modal motion and underscore the effectiveness of independent multi-chamber control in realizing complex spatial deformations.

**Figure 1:**
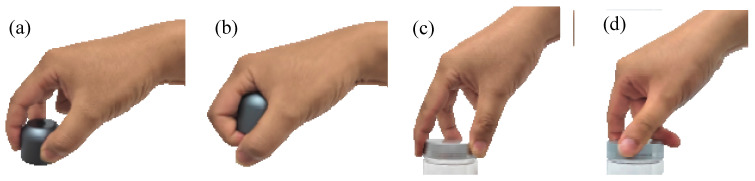
Hand grasping functions: (**a**) fingertip pinching; (**b**) enveloping grasping; (**c**,**d**) circular twisting.

As shown in Figure 1, human grasping operations include fingertip pinching, enveloping grasping, and circular twisting. However, reproducing the dexterous manipulation of the human hand—particularly fine operations such as “circular twisting” that require active object reorientation—remains a prominent challenge in soft grasping [18]. The dexterity of the human hand when grasping stems from the coordinated motion of multiple joints in bending and abduction/adduction DoFs. In recent years, researchers have made progress in replicating these DoFs on soft actuators through structural innovation. Xiaofeng et al. [19] bio-mimicked the skeletal structure of human fingers, designing a soft actuator with a stepped chamber structure (larger distal, smaller proximal) to enhance finger flexibility and increase output force. Wang Fujun et al. [20] designed a composite bending soft actuator mimicking distal bending and proximal abduction/adduction based on human finger motion. Cong Ming et al. [21] designed a three-joint soft actuator capable of highly mimicking the flexion-extension motion of human fingers. Chen Xuyang et al. [22] designed three-segment fingers and a thumb joint, creating a pneumatically driven soft robotic hand capable of performing basic gestures like “OK,” fist, and “peace.” Huadong et al. [23] adopted a modular design philosophy based on finger joint kinematics, manufacturing a highly adaptable, flexible soft glove composed of multi-modal deformable soft fingers. Jiralerspong et al. [24] and Yap et al. [25] used two independent soft actuators to support thumb flexion and abduction-adduction movements, respectively.

Nonetheless, existing research mostly focuses on replicating grasping modes themselves (e.g., pinching, enveloping) [26,27], or achieving complex motions through the combination of multiple actuators [28]. There remains a lack of systematic solutions for how to actively decouple and coordinate bending and abduction/adduction DoFs through the intrinsic structural design of a single actuator to reliably and controllably accomplish the composite task of “twisting.” The absence of this capability severely limits the practical application of soft grippers in scenarios requiring fine manipulation, such as assembly and maintenance.

Based on this, this paper proposes a four-chamber spatial bending soft actuator based on the principle of virtual work. The actuator features an internal “cross-shaped” restricting layer, forming four independently controllable pneumatic chambers. Its core innovation lies in enabling continuous bending deformation in any spatial direction by adjusting the pressure in each chamber, thereby simulating the bending and abduction/adduction DoFs of a human finger and their arbitrary combinations on a single actuator. The main contributions of this paper include: (1) Proposing a novel decoupled four-chamber actuator structure, providing a physical platform for achieving spatial bending and “twisting” manipulation. (2) Establishing a static deformation model of the actuator based on the Yeoh hyperelastic model and the principle of virtual work and proposing a simplified vector superposition method to predict deformation under non-uniform multi-chamber pressurization, enhancing model accuracy by introducing correction coefficients. (3) Constructing a three-finger soft gripper based on this actuator, achieving fingertip pinching and enveloping grasping. Systematically demonstrating the fine “circular twisting” operation (exemplified by light bulb installation) through open-loop programmed control, validating its potential in flexible grasping and fine manipulation. This study aims to explore the controllability of soft actuators in multi-DoF coupled motion through structural innovation and theoretical modeling, providing new ideas and design paradigms for soft robots performing complex tasks.

## 2. Soft Actuator Design

### 2.1. Bionic Principle

**Figure 2:**
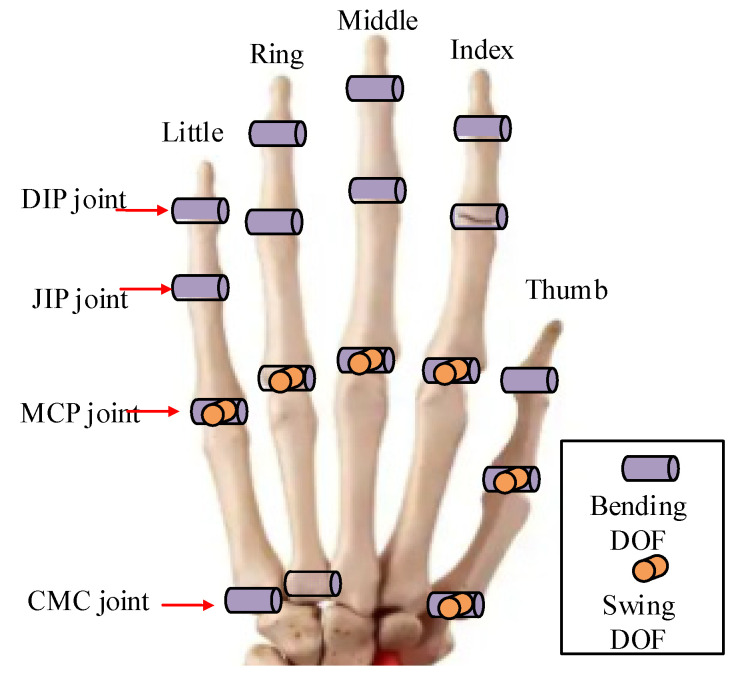
DOFs of human hands.

Anatomical studies generally agree [29,30] that the human hand possesses 23 degrees of freedom (excluding the wrist), as shown in Figure 2. Each finger (except the thumb) has 4 DoFs, including bending DoFs at 3 joints (Distal Interphalangeal joint, Proximal Interphalangeal joint, Metacarpophalangeal joint) and 1 abduction/adduction DoF at the Metacarpophalangeal joint. The little finger and ring finger also possess bending DoFs at the Carpometacarpal joint. The thumb has 5 DoFs, including bending DoFs at 3 joints (Interphalangeal joint, Metacarpophalangeal joint, Carpometacarpal joint) and abduction/adduction DoFs at 2 joints (Metacarpophalangeal joint, Carpometacarpal joint).

In the three-finger grasping operation shown in Figure 1, both fingertip pinching and enveloping grasping primarily utilize the bending DoFs of finger joints. In circular twisting, besides bending DoFs, the abduction/adduction DoF of the Metacarpophalangeal joint is also required to achieve object rotation. Inspired by this, this paper simplifies the bending DoFs of each finger into one primary bending DoF and retains the abduction/adduction DoF of the Metacarpophalangeal joint, thereby providing the basis for achieving circular twisting.

### 2.2. Structure Design of the Actuator

**Figure 3:**
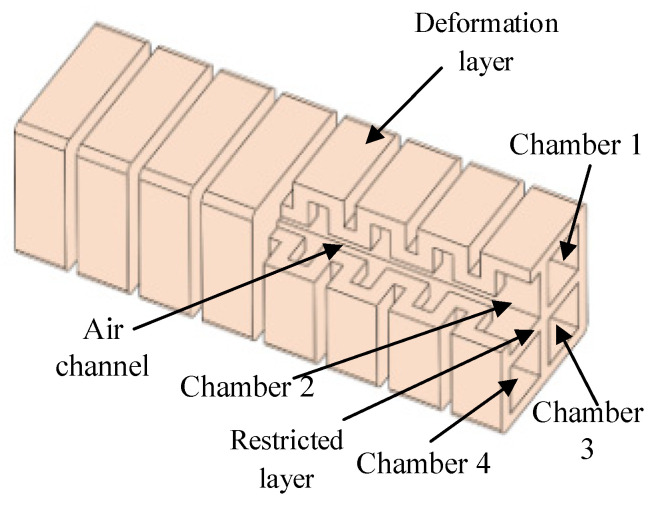
Soft actuator structure.

**Figure 4:**
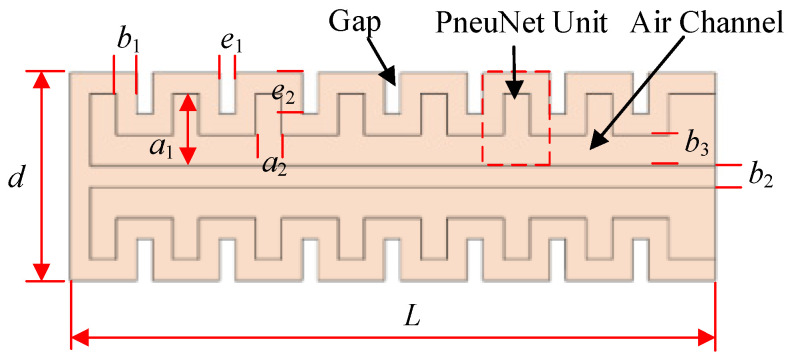
Schematic diagram of structural parameters.

Under the aforementioned bionic principle, it is necessary to realize independent bending and abduction/adduction deformations and their combined deformation on a single soft actuator (soft finger). The designed soft actuator (the actuator described in this paper can also be regarded as a bionic finger structure, hereafter uniformly referred to as ‘soft actuator’) structure is shown in Figure 3, featuring 4 independent pneumatic chambers. The soft actuator is overall a rectangular cuboid in cross-section, with an outer deformation layer. A “cross-shaped” restricting layer is set in the middle, dividing the internal space into four chambers. Each chamber consists of multiple grid cells, internally connected by air channels. Gaps are set between adjacent grid cells to increase the deformation magnitude and rate of the soft actuator. The internal structure and specific dimensions are shown in Figure 4 and Table 1.

**Figure 5:**
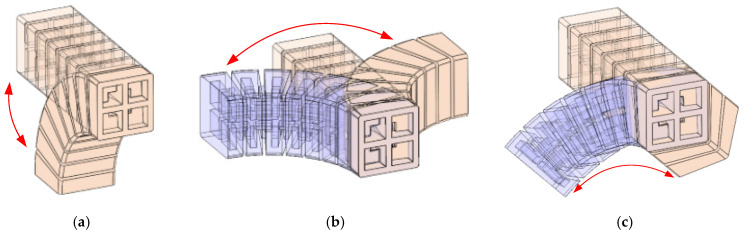
Soft actuator deformation modes of the soft actuator: (**a**) bending in the vertical plane; (**b**) bending in the horizontal plane; (**c**) combined deformation in an arbitrary spatial direction, resulting from the superposition of vertical and horizontal bending components.

With this structure, the four chambers are not interconnected, allowing independent control of gas inlet/outlet and pressure for each chamber. Under different chamber pressurization and different pressure levels, this soft actuator can produce various deformation modes in space. As shown in Figure 5, the actuator can produce three categories of deformation. When chambers 1 and 2 are pressurized and chambers 3 and 4 are vented, the actuator bends downward in the vertical plane, mimicking the bending (flexion) DoF of a human finger (Figure 5a). When chambers 1 and 3 are pressurized or chambers 2 and 4 are pressurized, the actuator bends leftward or rightward in the horizontal plane, mimicking the abduction/adduction DoF of the metacarpophalangeal joint (Figure 5b). Furthermore, by applying different pressures to all four chambers, the actuator can produce bending in any intermediate spatial direction. This combined deformation (Figure 5c) results from the simultaneous contribution of vertical and horizontal bending components, and can be continuously tuned by regulating the pressures in the four chambers.

### 2.3. Fabrication of the Soft Actuator

**Figure 6:**
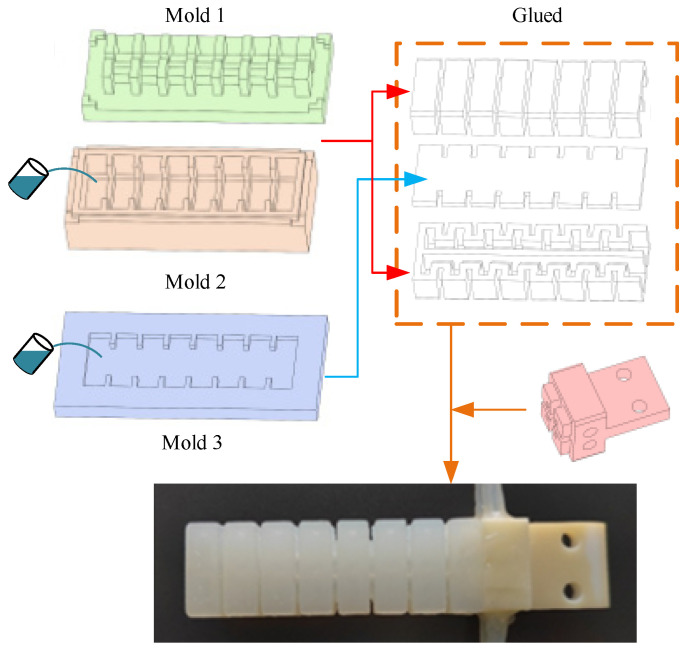
Steps for making soft fingers.

Casting is the primary method for fabricating silicone soft actuators. As illustrated in Figure 6, all molds used in the process were fabricated via 3D printing using PLA material. The fabrication procedure consisted of four main steps: (1) Casting two halves of the deformation layer using Mold 1 and Mold 2. (2) Casting the central restricting layer using Mold 3. (3) Bonding the two deformation layer halves with the central restricting layer. (4) Finally, attaching the connectors and air tubes to complete a soft actuator unit. The resulting fabricated prototype is shown in Figure 6.

The soft fingers were made of a commercial silicone rubber (Dongguan Aocai New Materials Co., Ltd., Dongguan China Shore hardness 20A). The material constants, determined by uniaxial tensile tests, were C_10_ = 0.16 and C_20_ = 0.02, as used in the subsequent Yeoh hyperelastic model. The detailed structural parameters of the soft fingers are provided in Table 1.

## 3. Finite Element Analysis and Mathematical Modeling

This section aims to establish a static deformation theoretical model for the described four-chamber soft actuator to reveal the quantitative mapping relationship between “multi-chamber pressure input—spatial bending output” and guide subsequent precise control. First, key characteristics of its deformation and internal stress distribution are analyzed through finite element simulation, based on which core assumptions for theoretical modeling are proposed. Then, the theoretical model is derived based on the principle of virtual work.

**Figure 7:**
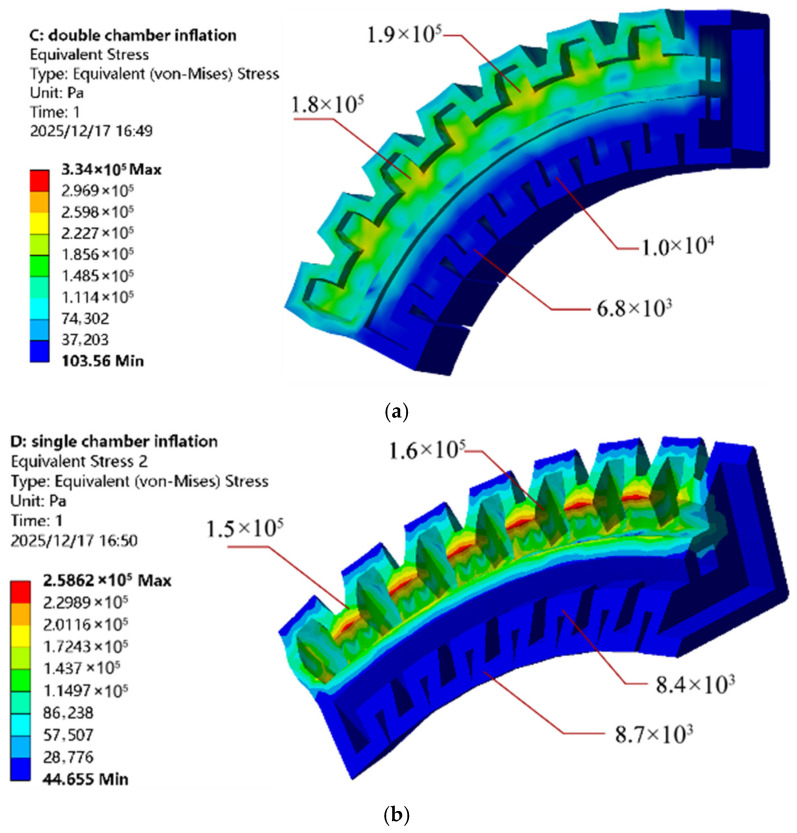
Stress distribution simulation results: (**a**) dual-chamber inflation: 100 kPa in chambers 1 and 2; (**b**) single-chamber inflation: 100 kPa in chamber 1.

The four-chamber structure of the actuator is designed to achieve decoupled motion through independent pressure control. To verify this design effect, we first analyzed the internal stress distribution under single-chamber and dual-chamber inflation (Figure 7, stress distribution simulation results). The simulation results indicate that when the inflated chamber(s) (e.g., chamber 1 or chambers 1 and 2) expand, stress is highly concentrated in the inflated region, with typical values in the range of 100–200 kPa. Due to the presence of structural gaps, the internal stress in adjacent non-inflated chambers is significantly lower, with typical values below 10 kPa, nearly an order of magnitude difference from the stress in the inflated chambers. This phenomenon implies that the hindering effect (reaction force) of non-inflated chambers on overall deformation is much smaller than the active driving effect of the inflated chambers. Meanwhile, the stress distribution within each inflated grid cell is relatively uniform.

This effect stems from the gap design between deformation layers. When an inflated chamber expands, non-inflated chambers are compressed, and the gaps absorb most of the deformation, resulting in minimal deformation of the silicone in non-inflated chambers and consequently very low internal stress, having little impact on the overall deformation of the soft actuator. Simultaneously, the small coupling effect between chambers also indicates that chamber inflation deformation is relatively independent. Based on this, we propose two core assumptions for this theoretical modeling: (1) Neglect the reaction of non-inflated chambers. Given that their stress is much lower than that of inflated chambers, the elastic restoring force generated by material deformation in non-inflated chambers can be ignored in force balance analysis. (2) Deformation is linearly superimposable. Owing to the weak stress coupling between chambers, the composite deformation of the four chambers under non-uniform pressure can be approximated as the linear superposition of the deformation vectors produced by inflating each chamber independently.

Subsequent theoretical derivation will be based on the above assumptions. First, two basic working conditions—single-chamber inflation and dual symmetric-chamber inflation—are analyzed to establish the analytical relationship between pressure and bending angle (Section 2.1 and Section 2.2). Then, based on the superposition assumption, a spatial bending prediction model under arbitrary pressure input for four chambers is derived (Section 2.3).

**Figure 8:**
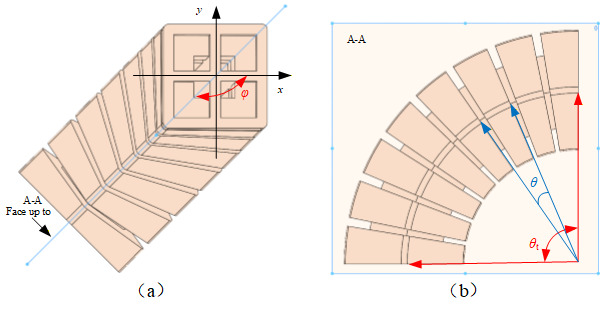
Definition of bending deformation parameters: (**a**) front view, the direction is the angle between the deformation plane A-A and the horizontal axis; (**b**) face up to plane A-A.

As shown in Figure 8a, define the bending direction as *φ* (the angle between the bending plane and the horizontal axis). As shown in Figure 8b, the bending angle corresponding to a single PneuNet is *θ*, and the total bending angle is *θ*_t_.

Therefore, the theoretical analysis aims to establish a vector-valued function that maps the four chamber pressures to the bending parameters, i.e.,

(1) $$\left(\varphi ,\theta_{t}\right)=f\left(p_{1},p_{2},p_{3},p_{4}\right)$$

Assuming the central angle corresponding to each PneuNet remains the same, the overall bending central angle of the soft actuator can be considered as the sum of the bending central angles contributed by each PneuNet, i.e.,

(2) $$\theta_{t}=n\theta$$

where *n* is the number of PneuNets in the soft actuator, *n* = 7.

The expression for the two-parameter Yeoh model is given by Equation (3), and by taking the axial elongation as the principal elongation, the relationship between stress and principal elongation ratio can be derived as Equation (4) [5,15].

(3) $$\begin{array}{l} W=C_{10}\left(I_{1}-3\right)+C_{20}{\left(I_{2}-3\right)}^{2}\\ =C_{10}{\left(\lambda_{1}-\frac{1}{\lambda_{1}}\right)}^{2}+C_{20}{\left(\lambda_{1}-\frac{1}{\lambda_{1}}\right)}^{4} \end{array}$$

(4) $$\sigma =2\lambda_{1}\left(1-\frac{1}{{\lambda_{1}}^{4}}\right)\left[C_{10}+2C_{20}{\left(\lambda_{1}-\frac{1}{\lambda_{1}}\right)}^{2}\right]$$

where *W* is the strain energy function, *I*_i_ are the first and second invariants of the deformation tensor, *σ* is strain, *λ*_1_ is the principal elongation ratio, C_10_ and C_20_ are material coefficients of the silicone obtained through uniaxial tensile tests, C_10_ = 0.16, C_20_ = 0.02.

### 3.1. Deformation and Simulation for Two Chambers Inflated

**Figure 9:**
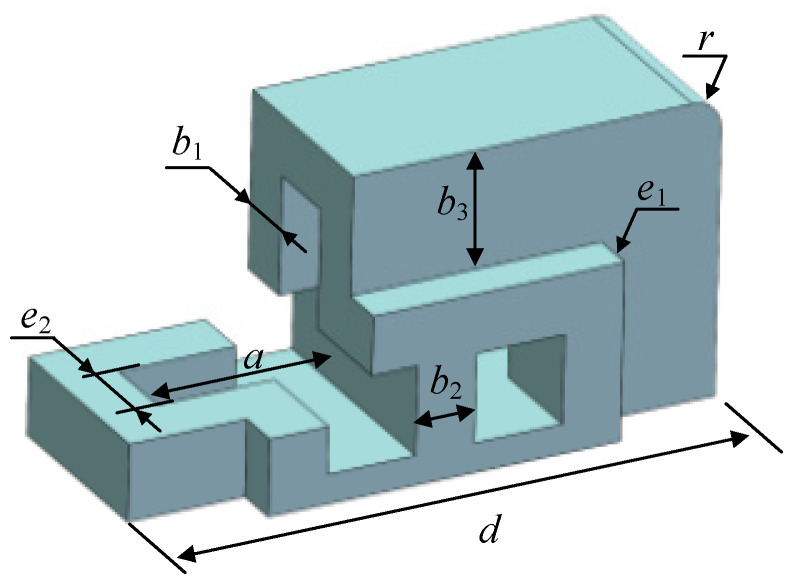
PnueNet structure.

Analyze the bending deformation situation when two chambers on the same side are inflated with the same pressure. Assume chambers 1 and 2 are connected to the same pressure, and chambers 3 and 4 are directly connected to the atmosphere. At this point, chambers 1 and 2 expand and deform, causing the entire soft actuator to bend towards the side of chambers 3 and 4, i.e., $\varphi_{12}=-90{}^{\circ}$. Taking one PnueNet of chambers 1 and 2 for analysis, its cross-section is shown in Figure 9.

According to the principle of virtual work:

(5) $$p_{12}dV_{q12}=V_{g12}dW$$

where *p*_12_ is the internal gas pressure in chambers 1 and 2, *V_g_*_12_ is the volume of the silicone material in a single PnueNet, and *V_q_*_12_ is the gas volume within the PnueNet.

Assuming the silicone material is incompressible, its volume remains unchanged before and after deformation. The gas volume after deformation is:

(6) $$V_{q12}=V_{12}-V_{g12}={V_{12}}^{0}\left(1+\lambda \right)-V_{g12}$$

where *V*_12_ is the total volume of the single PnueNet after deformation and *V*_12_^0^ is the initial volume of the PnueNet before deformation. *λ* represents the axial elongation ratio of PnueNet. Assuming the middle restriction layer does not elongate, taking the elongation of the unit’s central axis as the principal axial elongation, the principal elongation ratio can be expressed as:

(7) $$\lambda^{\prime \prime }=\frac{h+\theta_{12}d/4}{h}$$

where h is the PnueNet thickness, $h=e_{1}+a_{2}+2b_{1}$. *θ*_12_ is the central angle corresponding to the single PnueNet.

Differentiating both sides of Equation (5) with respect to *θ*_12_ yields:

(8) $$p_{12}\frac{dV_{q12}}{d\theta_{12}}=V_{g12}\frac{dW}{d\theta_{12}}$$

(9) $$\frac{dV_{q12}}{d\theta_{12}}={V_{12}}^{0}\frac{d\lambda^{\prime \prime }}{d\theta_{12}}$$

(10) $$\frac{dW}{d\theta_{12}}=\frac{dW}{d\lambda^{\prime \prime }}\frac{d\lambda^{\prime \prime }}{d\theta_{12}}=\sigma \left(\lambda^{\prime \prime }\right)\frac{d\lambda^{\prime \prime }}{d\theta_{12}}$$

Substituting Equations (9) and (10) into Equation (8), resulting in:

(11) $$p_{12}=\frac{V_{g12}}{{V_{12}}^{0}}\sigma \left(\lambda^{\prime \prime }\right)=f\left(\theta_{12}\right)$$

In Equation (7), $\lambda^{\prime \prime }$ can be expressed as a function of *θ*_12_ only. Therefore, Equation (11) contains only two variables, *p*_12_ and *θ*_12_. Once the driving pressure is known, the corresponding bending angle for a single PnueNet can be calculated. Then, the overall bending angle of the soft actuator can be calculated according to Equation (2).

(12) $$\theta_{t12}=nf^{-1}\left(p_{12}\right)$$

Expressing the deformation angle and direction of the soft actuator as a vector gives:

(13) $$\theta_{t\_12}=\theta_{t12}e^{j\varphi_{12}}=nf^{-1}\left(p_{12}\right)e^{-90{}^{\circ}j}$$

The bending deformation relational expressions for the other three same-side inflation cases can be obtained similarly.

**Figure 10:**
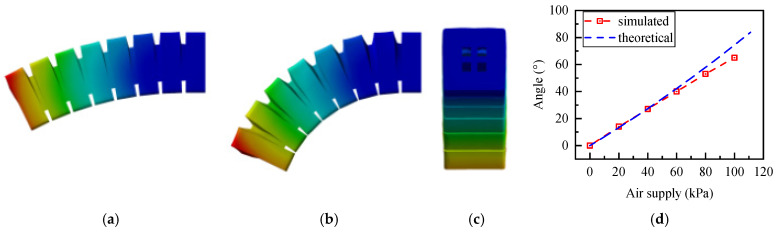
Dual-chamber inflation deformation results: (**a**) $p_{1}=p_{2}=20\;\mathrm{kPa}$; (**b**) $p_{1}=p_{2}=100\;\mathrm{kPa}$; (**c**) $p_{1}=p_{2}=100\;\mathrm{kPa}$, front view; (**d**) Calculation and simulation results.

Importing the soft actuator into finite element software Workbench, setting the same pressure in chambers 1 and 2 (*p*_1_ = *p*_2_), and connecting chambers 3 and 4 to the atmosphere (*p*_3_ = *p*_4_ = 0), the deformation of the soft actuator under different supply pressures is as shown in Figure 10. Plotting the simulation results and theoretical calculation results in Figure 10d shows that when the pressure is less than 40 kPa, the calculated value is basically consistent with the simulated value. As the pressure increases, the calculated value is greater than the simulated value. When the pressure is 100 kPa, the simulated bending angle is 65°. And the calculated bending angle is 74.5°.

### 3.2. Deformation and Simulation for Single Chamber Inflation

Assume chamber 1 is connected to compressed gas, and chambers 2, 3, and 4 are directly open to the atmosphere. At this point $\varphi_{1}=-135{}^{\circ}$. The virtual work equation is:

(14) $$p_{1}dV_{q1}=V_{g1}dW$$

where *p*_1_ is the internal gas pressure in chamber 1, *V_g_*_1_ is the volume of the silicone material in a single PnueNet of chamber 1, and *V_q_*_1_ is the gas volume within the PnueNet. The gas volume after deformation is:

(15) $$V_{q1}=V_{1}-V_{g1}=V_{1}^{0}\left(1+\lambda \right)-V_{g1}$$

where *V*_1_ is the total volume after deformation and *V*_1_^0^ is the initial volume before deformation. Again, taking the elongation of the chamber’s central axis as the axial elongation, the principal elongation ratio at this time is:

(16) $$\lambda^{\prime }=\frac{h+\theta_{1}d\sqrt{2}/4}{h}$$

where *θ*_1_ is the central angle corresponding.

Differentiating both sides of Equation (14) with respect to *θ*_1_ yields:

(17) $$p_{1}=\frac{V_{g1}}{V_{1}^{0}}\sigma \left(\lambda^{\prime }\right)=g\left(\theta_{1}\right)$$

Similarly, the deformation of the soft actuator can be expressed as:

(18) $$\theta_{t\_1}=\theta_{t1}e^{j\varphi_{1}}=ng^{-1}\left(p_{1}\right)e^{j135{}^{\circ}}$$

The cases for the other three chambers inflated individually can be obtained similarly.

**Figure 11:**
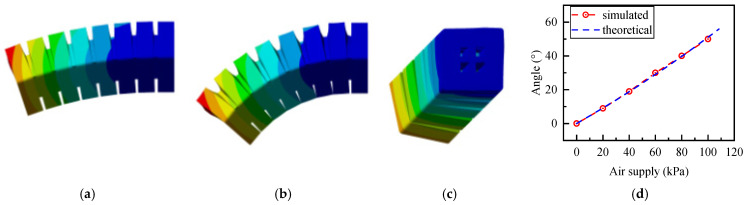
Single-chamber inflation deformation results: (**a**) $p_{1}=20\;\mathrm{kPa}$; (**b**) $p_{1}=100\;\mathrm{kPa}$; (**c**) $p_{1}=100\;\mathrm{kPa}$, front view; (**d**) calculation and simulation results.

In Workbench, setting the pressure *p*_1_ in chamber 1, and connecting chambers 2, 3, 4 to the atmosphere (*p*_2_ = *p*_3_ = *p*_4_ = 0), the deformation of the soft actuator under different supply pressures is as shown in Figure 11. Plotting the simulation results and theoretical calculation results again shows that the error between the two is extremely small, and the two curves basically overlap.

### 3.3. Empirical Formula for Four-Chamber Inflation Deformation

Based on the basic models established in Section 3.1 and Section 3.2 and the aforementioned superposition assumption, the overall bending deformation vector of the four chambers under any pressure combination $\left(p_{1},p_{2},p_{3},p_{4}\right)$ can be approximated by the superposition of contributions from individual chambers. That is, the non-uniform pressure field of the four chambers is equivalently decomposed into several symmetric pressure fields, which is a simplified engineering method based on single/dual-chamber models. The expression is:

(19) $$\theta_{t}=\theta_{t}e^{j\varphi }=\sum \theta_{t\_\mathrm{mn}}+\sum \theta_{t\_i}$$

where ***θ*****_t_mn_** is the bending vector produced by same-side dual-chamber inflation (calculated by Equation (12)), and ***θ*****_t_i_** is the bending vector produced by single-chamber inflation (calculated by Equation (18)).

**Figure 12:**
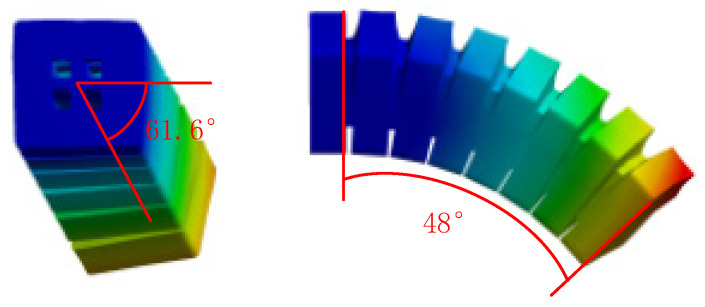
Four-chamber inflation deformation simulation results.

For example, when the four-chamber pressures are $\left(p_{1},p_{2},p_{3},p_{4}\right)=\left(70,100,0,40\right)$ kPa (as shown in Figure 12, the deformation direction is −61.6° and the bending angle is 48°), it can be decomposed as the vector sum of deformations from chambers 1 and 2 under 70 kPa, chambers 1 and 4 under 30 kPa, and chamber 4 under 10 kPa, expressed as Equation (20). The calculated deformation direction is −63.4° and the bending angle is 52.3°. Compared to the simulation results, the error of deformation direction is approximately 3% and the error of bending angle is approximately 8%. The error is small.

(20) $$\begin{array}{l} \theta_{t}=\theta_{t}e^{j\varphi_{t}}=\theta_{t\_12}+\theta_{t\_24}+\theta_{t\_4}\\ =nf^{-1}\left(70\right)e^{-90{}^{\circ}j}+nf^{-1}\left(30\right)e^{j0{}^{\circ}}+ng^{-1}\left(10\right)e^{j45{}^{\circ}}\\ =52.3e^{-63.4{}^{\circ}j} \end{array}$$

## 4. Soft Actuator Characteristic Experiments

**Figure 13:**
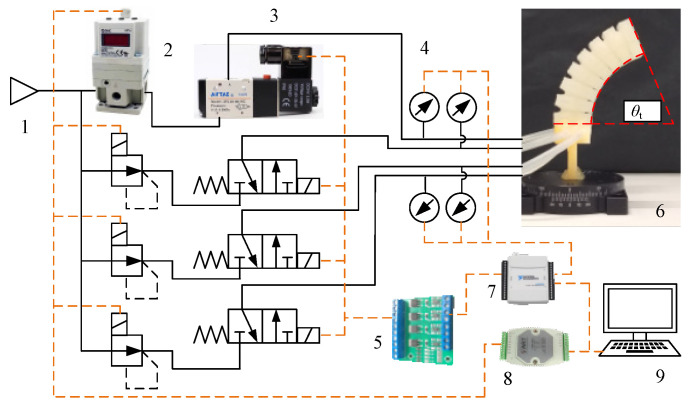
Schematic diagram of experiment platform: 1—air source; 2—pressure proportional valve (SMC: ITV1110); 3—solenoid valve (AirTAC: 3V110); 4—pressure gauge (SMC: ISE30A); 5—driver board; 6—rotating table; 7—DAQ card (NI: USB-6009); 8—D/A converter (ART Technology: DAM-3060); 9—computer.

To verify the accuracy of the theoretical model and evaluate the actuator performance, this section conducts static and dynamic characteristic experiments. Using the fabricated soft actuator, an experimental platform is constructed as shown in Figure 13, where a rotary stage is placed horizontally, the soft actuator is vertically installed at the center of the stage, and a camera is set directly in front. After the soft actuator fully deforms, photos are taken to record the bending shape, and bending angle data is extracted in image processing software (ignoring inevitable perspective effects during shooting).

### 4.1. Static Characteristics

**Figure 14:**
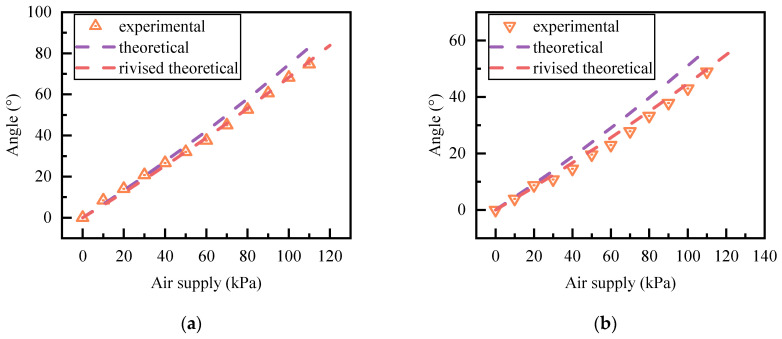
Dual-chamber and single-chamber inflation experimental results: (**a**) Dual-chamber inflation experimental results. (**b**) Single-chamber inflation experimental results.

**Figure 15:**
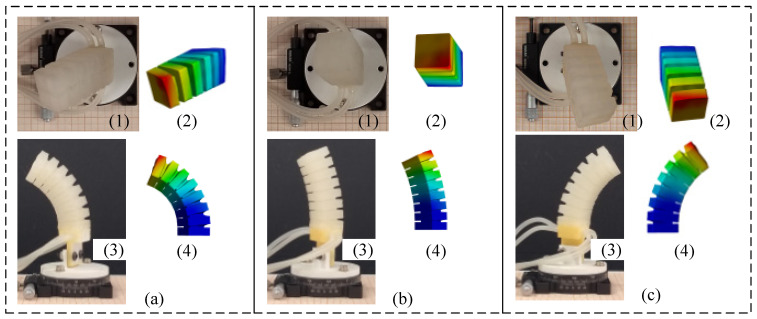
Comparison experimental results with simulation results: (1) and (3): experimental results, vertical view and front view; (2) and (4): simulation results; (**a**) $\left(p_{1},p_{2},p_{3},p_{4}\right)=\left(20,130,0,70\right)$ kPa; (**b**) $\left(p_{1},p_{2},p_{3},p_{4}\right)=\left(30,50,0,20\right)$ kPa; (**c**) $\left(p_{1},p_{2},p_{3},p_{4}\right)=\left(80,100,20,0\right)$ kPa.

Chambers 1 and 2 on the same side and single chamber 1 are inflated with compressed gas from 0 to 100 kPa, measuring the deformation angles under dual-chamber and single-chamber inflation. The experimental data and theoretical calculation values are plotted in Figure 14. It can be seen that in both cases, the theoretical values are larger than the experimental values, and the error increases with increasing supply pressure. In the dual-chamber inflation experiment, the error between theoretical calculation and experimental values is relatively small. When the supply pressure is 100 kPa, the experimentally measured bending angle is 68.2°, and the theoretically calculated bending angle is 74.5°, with an error of about 8.5%. In the single-chamber inflation experiment, the error is larger. When the supply pressure is 100 kPa, the experimentally measured bending angle is 43°, and the theoretically calculated bending angle is 50.8°, with an error reaching 15.3%. The reason is that we neglected the reaction of non-inflated chambers in theoretical modeling. Since stress simulation showed the stress in non-inflated chambers is very small, the theoretical value is larger than the experimental result. For dual-chamber inflation, two non-inflated chambers are neglected, while for single-chamber inflation, three non-inflated chambers are neglected, hence the larger error. To reduce the error between theory and experiment, correction coefficients are introduced, modifying Equations (11) and (17) to Equation (21), and the values of the correction coefficients are determined by fitting experimental data (k_2_ = 1.08, k_1_ = 1.19). As shown by the red curve in Figure 15, the corrected theoretical curve closely matches the experimental data.

(21) $$\left\{\begin{array}{l} p_{12}=k_{2}\frac{V_{g12}}{{V_{12}}^{0}}\sigma \left(\lambda^{\prime \prime }\right)=f\left(\theta_{12}\right)\\ p_{1}=k_{1}\frac{V_{g1}}{{V_{1}}^{0}}\sigma \left(\lambda^{\prime }\right)=g\left(\theta_{1}\right) \end{array}\right.$$

Finally, experiments with non-uniform inflation of all four chambers are conducted. Two sets of input pressures are randomly selected (Table 2), with the corresponding experimental photographs and simulation results shown in Figure 15. The experimentally measured results are compared with the simulation results and the corrected theoretical superposition calculation results (Table 2). It can be observed that the corrected pressure-deformation prediction model agrees well with the experimental data, with an error of only about 3%. This indicates that the method of equivalently decomposing the non-uniform pressure field of the four chambers into the linear superposition of several symmetric pressure fields is effective in this study. It enables effective prediction of deformation direction and angle in open-loop control through straightforward calculations using Equation (19).

### 4.2. Dynamic Characteristics

**Figure 16:**
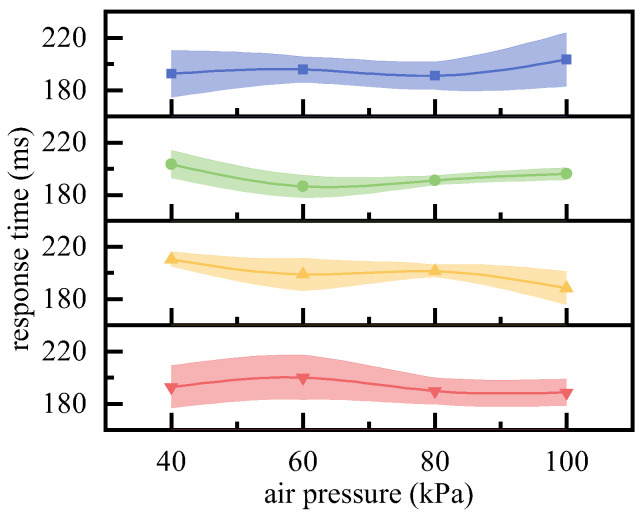
Response to experimental results.

The response time is defined as the duration required for the pressure reading of pressure gauge 2 to rise from 0 to the stable set value after the valve is activated. Tests were conducted incrementally within a supply pressure range of 40 kPa to 100 kPa. Each pressure level was tested three times, and the average value was taken as the final result. The results plotted in Figure 16 show that the soft actuator’s response time fluctuates minimally within the tested pressure range, with an average response time of 195 milliseconds.

## 5. Soft Actuator Application

**Figure 17:**
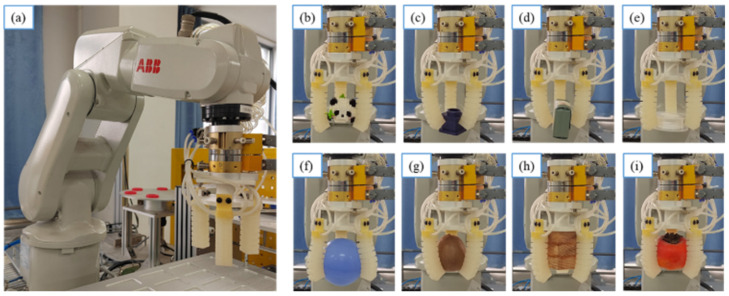
Enveloping grasping and fingertip pinching experiments: (**a**) robotic arm with the three-finger soft gripper; (**b**–**e**) fingertip pinching: building blocks, aluminum alloy part, tea bag, and tape roll; (**f**–**i**) enveloping grasping: ocean ball, kiwifruit, wooden block, and persimmon.

To mimic human finger functions such as fingertip pinching, enveloping grasping, and circular twisting, a three-finger human-hand-inspired multifunctional soft gripper is designed. Three soft actuators are uniformly distributed circumferentially on a palm component. The soft actuator connectors are rigid parts (resin material, 3D printed), while the rest are flexible parts (silicone material, cast using molds). The palm is a rigid part (resin material, 3D printed), with a top connecting flange for attachment to a robotic arm end-effector (ABB robot, model: IRB120), as shown in Figure 17a.

### 5.1. Demonstration of Enveloping Grasping and Fingertip Pinching Functions

Selecting small-volume, thin objects like building blocks, CNC aluminum alloy parts, tea bags, and tape rolls, fingertip pinching tests are performed using the fingertip portion of the soft actuator. Selecting larger-volume objects like ocean balls, kiwifruit, wooden blocks, and persimmons for enveloping grasping tests. The results are shown in Figure 17b–i. The soft gripper can stably grasp the aforementioned test objects and maintain the gripping state without dropping during robotic arm movement. This demonstrates that the soft gripper can accomplish enveloping grasping and fingertip pinching functions.

### 5.2. Circular Twisting Experiment—Bulb Installation

To verify the intelligence and programmability of the actuator in performing complex manipulation tasks, we designed a “light bulb installation” task as a proof of concept. This task requires the actuator to execute a continuous rotational motion trajectory after grasping the bulb to screw it into the socket.

**Figure 18:**
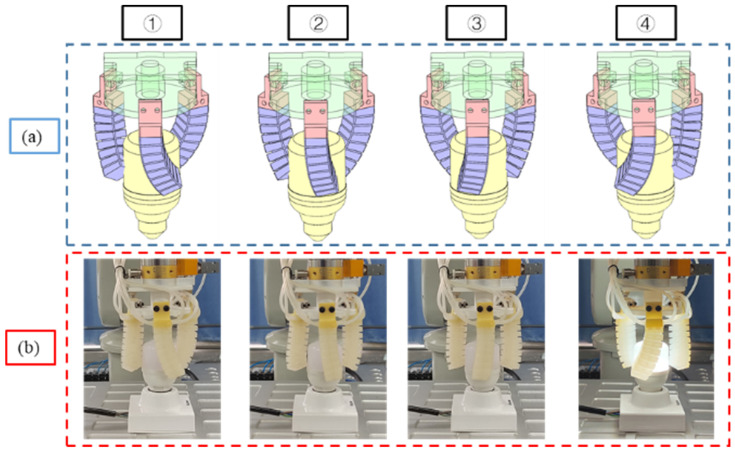
Circular twisting function experiment: rotating to install and light a bulb. Bulb specifications: diameter 50 mm, height 97 mm, screw base E27, power 5 W.: (**a**) 3D model of soft gripper and light bulb; (**b**) installation process.

We first discretized this continuous trajectory into four key operational poses (Figure 18a). Through 3D modeling, the spatial positions required for the actuator’s fingertip in each pose were precisely determined, thereby deriving the target bending direction angle *φ* and bending angle *θ*_t_ for the actuator body (Table 3). Subsequently, based on the corrected theoretical model and Equation (19), the precise four-chamber pressure combinations required to achieve each target pose were inversely solved. This process essentially maps the trajectory in task space to the actuator’s pressure control space. Finally, we obtained a time-sequenced set of pressure control command sequences (Table 3). In the experiment, simply inputting this pre-programmed sequence to the actuator drives it to automatically complete the entire twisting operation.

As shown in Figure 18b, the actuator was strictly executed according to the pre-programmed pressure command sequence, successfully completing the entire operation flow from initial grasping, continuous rotation, to final tightening, and successfully lighting the bulb. This result holds dual significance: firstly, it directly validates the effectiveness and practicality of the theoretical model from Section 3 in inverse problem solving. More importantly, it proves that our proposed four-chamber actuator can serve as a reliable motion execution end-effector, faithfully reproducing complex spatial motion trajectories planned by upstream algorithms (or operators).

Table 3 clearly shows the mapping relationship from high-level task objectives (pose *φ*, *θ*_t_) to low-level driving commands (*p*_1_–*p*_4_). This deterministic mapping is an indispensable foundation for soft actuators to achieve higher-level intelligent closed-loop control (e.g., combined with visual feedback).

## 6. Discussion

The four-chamber actuator proposed in this study and its theoretical model demonstrate potential in achieving human-hand-like fine manipulation. The following discussion comprehensively addresses the engineering approach of model construction, the significance of the open-loop control demonstration, and the system’s limitations, to clarify the contributions of this work and future improvement directions.

The core of the theoretical model in this paper lies in the two assumptions proposed based on observations from finite element simulation (Figure 7): “neglecting the reaction of non-inflated chambers” and “deformation vector superposition.” Within the experimental parameter range (pressure ≤ 100 kPa, bending angle ≤ 70°), these assumptions achieve a good balance between simplicity and accuracy. However, it must be recognized that the engineering applicability of these assumptions has clear boundaries.

The reasonableness of “neglecting the reaction of non-inflated chambers” highly depends on the structural design of the inter-chamber gaps (e_1_, e_2_). The gap dimensions in this study effectively absorb compression deformation on the non-inflated side, reducing its stress by an order of magnitude compared to the inflated chambers. If the gaps are too small or zero, mechanical coupling between chambers will increase, and forced compression of non-inflated chambers will generate non-negligible elastic reaction forces, introducing significant error if neglected. Conversely, excessively large gaps may weaken the overall structural stiffness of the actuator. “Deformation vector superposition” is essentially a linear approximation. When driving pressure causes extremely large strains, strong nonlinearities in material and geometry as well as deformation interference between chambers may cause the superposition principle to fail. Therefore, this model is most suitable for actuators with sufficiently decoupled structures working within a moderate deformation range. For designs operating beyond this range or with different structures, finite element methods or more complex coupled models are needed for prediction.

To bridge the gap between the simplified model and real complex behavior, we introduced empirical correction coefficients k_1_ and k_2_ obtained through experimental data fitting. Such coefficients effectively compensate for systematic errors caused by unmodeled factors (such as material parameter deviations, manufacturing inconsistencies, and weak inter-chamber coupling), significantly improving the model’s prediction accuracy for a specific actuator. However, the coefficient values depend on specific geometry, materials, and processes. Therefore, when applying this model to different instances, the coefficients need recalibration. This reveals the challenge the current method faces in pursuing generality. Future work needs to explore more fundamental, physics-based compensation mechanisms.

The “light bulb twisting” experiment successfully demonstrated open-loop programmed control based on the inverse solution of the theoretical model. This verifies the feasibility of mapping high-level tasks (spatial trajectories) to low-level driving commands (chamber pressures), marking a step in control strategy from basic deformation towards task-oriented motion synthesis. Although currently open-loop, this demonstration is crucial: it indicates that such decoupled-design actuators can serve as reliable execution end-effectors, reproducing planned complex motions. This lays a solid platform foundation for future integration of real-time visual or tactile feedback to build adaptive closed-loop control systems. The pressure sequence shown in Table 3 can be regarded as programmable motion primitives for achieving “circular twisting.”

To position this work within the current state of the art, a comparison with recent representative soft gripper actuators is provided in Table 4. Compared with these designs [5,6,10,13,19,20], the proposed actuator offers two distinguishing features: (1) the integration of bending and abduction/adduction DoFs within a single uniform four-chamber structure via structural decoupling, avoiding modular assembly or segmented design; and (2) a virtual-work-based analytical model with dual correction coefficients that enables deterministic inverse mapping from task space to actuation space, providing a foundation for programmed dexterous manipulation such as the demonstrated circular twisting task.

It should be noted that the actuator structure presented here is related to a previously reported four-chamber design by our group [31], which employed a moment balance model with a unified correction coefficient. The present study extends that work by adopting the virtual work principle for model derivation and introducing mode-specific correction coefficients, thereby improving prediction accuracy and enabling model-based inverse control—advances that were not addressed in the prior publication.

Future work can proceed in the following directions: continue exploring more accurate material models and attempt to establish a theoretical framework incorporating inter-chamber coupling terms to expand the model’s applicability and predictive capability; conduct parametric study and optimal design of key geometric parameters such as gap dimensions and chamber shapes, aiming for the best balance between decoupling performance, output force, and structural stiffness; build upon the current open-loop programmed control by introducing real-time sensor feedback to develop adaptive, compliant closed-loop control algorithms capable of handling object pose uncertainty and environmental disturbances; and evaluate and enhance the actuator’s load capacity, durability, and response speed to meet the demands of broader practical application scenarios.

## 7. Conclusions

Aiming at the challenge for soft grippers to achieve fine manipulation akin to human fingers, this study proposed a spatial bending soft actuator based on a four-chamber decoupled design. Through internal cross-shaped restricting layers and independent air path control, the actuator achieved active decoupling of bending and abduction/adduction degrees of freedom and continuous controllable bending in any spatial direction. Based on the Yeoh hyperelastic constitutive model and the principle of virtual work, combined with the engineering assumption of “deformation vector superposition,” a static prediction model from multi-chamber pressure input to spatial bending output was established. This model was corrected using experimental data fitting, providing a reliable theoretical inverse mapping for open-loop control of the actuator.

The three-finger soft gripper constructed based on this actuator not only achieved stable enveloping grasping and fingertip pinching of objects with various shapes and sizes but also systematically demonstrated the complex human-like fine operation of “circular twisting” (exemplified by light bulb installation) through pre-programmed pressure sequences. This validates the capability of this actuator as a programmable motion execution end-effector.

This work demonstrates that through structural decoupling design and corresponding simplified modeling methods, multi-degree-of-freedom motion can be effectively integrated into a single soft actuation unit, achieving deterministic mapping from task space to actuation space. This takes a key step towards developing adaptive soft grasping systems capable of autonomously switching grasping modes based on objects and tasks. In future work, integrating real-time visual or tactile feedback with intelligent algorithms on this hardware platform and control framework could enable truly dexterous and safe manipulation of complex, fragile objects in unstructured environments.

## Figures and Tables

**Table 1:** Structural dimensions.

Symble	Describtion	Value (mm)
*a*_1_	Length of PneuNet	7
*a*_2_	Width of PneuNet	2.5
*b*_1_	Thickness of deformation layer	2
*b*_2_	Thickness of restricted layer	2
*b*_3_	Width of air channel	3
*e*_1_	Width of gap	1.5
*e*_2_	Depth of gap	4
*L*	Length of actuator	62.5
*d*	Width of actuator	20

**Table 2:** Comparison of deformation results under arbitrary supply pressures.

	Experiment a: $\left(p_{1},p_{2},p_{3},p_{4}\right)=\left(20,130,0,70\right)$		Experiment b: $\left(p_{1},p_{2},p_{3},p_{4}\right)=\left(30,50,0,20\right)$		Experiment c: $\left(p_{1},p_{2},p_{3},p_{4}\right)=\left(80,100,20,0\right)$	
	Direction *φ*	Angle *θ_t_*	Direction *φ*	Angle *θ_t_*	Direction *φ*	Angle *θ_t_*
Experimental	−28.4°	60°	52.4°	25.5°	−107.8°	57.9°
Simulation	−26.2°	67.4°	54°	23.8°	−104.9°	53.5°
Calculated	−22.3°	61.7°	56.5°	22.3°	−103°	54.6°

**Table 3:** Bending directions and angles.

Seq.	Direction *φ*, Angle *θ_t_*	Pressures *p*_1_, *p*_2_, *p*_3_, *p*_4_ (kPa)
0	0°, 0°	0, 0, 0, 0
1	30°, 50°	70, 125, 0, 55
2	70°, 45°	70, 95, 0, 25
3	110°, 45°	95, 70, 25, 0
4	150°, 50°	125, 70, 55, 0

**Table 4:** Comparison of the proposed actuator with recent representative soft finger-like actuators.

Study	Actuator Type	Degrees of Freedom	Bending Capability
Cai et al. [5]	Dual-chamber composite (root + tip)	1 DoF (planar bending per segment)	Planar bending
Guo et al. [6]	Oblique-chamber spiral with stiffener panels	Spiral bending (fixed trajectory)	Helical enveloping
Li et al. [10]	Multi-structure (PneuNet proximal + NSPA distal)	1 DoF (planar bending)	Planar bending
Liu and Wang [13]	Two-module DMVCHA (variable chamber height)	1 DoF per module (planar bending)	Planar bending
Yu et al. [19]	Stepped chamber + rigid-flexible coupling	2-DoF (two segments, independently actuated)	Planar bending
Wang et al. [20]	Multi-chamber composite (single + dual chambers)	Forward + lateral bending	Bending in two orthogonal planes
This work	Four-chamber with cross-shaped restricting layer	Bending + abduction/adduction in a single uniform structure	Continuous bending in arbitrary spatial directions

## Data Availability

All data generated or analyzed during this study are included in this article.
